# The Expression of Affective Temperaments in Cystic Fibrosis Patients: Psychopathological Associations and Possible Neurobiological Mechanisms

**DOI:** 10.3390/brainsci13040619

**Published:** 2023-04-05

**Authors:** Andrea Amerio, Luca Magnani, Carlo Castellani, Irene Schiavetti, Gabriele Sapia, Francesca Sibilla, Rita Pescini, Rosaria Casciaro, Federico Cresta, Andrea Escelsior, Alessandra Costanza, Andrea Aguglia, Gianluca Serafini, Mario Amore, Riccardo Ciprandi

**Affiliations:** 1 Department of Neuroscience, Rehabilitation, Ophthalmology, Genetics, Maternal and Child Health (DINOGMI), Section of Psychiatry, University of Genoa, 16132 Genoa, Italy; gabriele.sapia93@gmail.com (G.S.); andrea.escelsior@live.com (A.E.); andrea.aguglia@unige.it (A.A.); gianluca.serafini@unige.it (G.S.); mario.amore@unige.it (M.A.); 2 IRCCS Ospedale Policlinico San Martino, 16132 Genoa, Italy; francesca.sibilla@hsanmartino.it; 3 Department of Mental Health and Pathological Addictions, Genoa Local Health Authority, 16126 Genoa, Italy; magnani1991@gmail.com; 4 Cystic Fibrosis Center, IRCCS Istituto Giannina Gaslini—Ospedale Pediatrico, 16147 Genoa, Italy; carlocastellani@gaslini.org (C.C.); ritapescini@gaslini.org (R.P.); rosariacasciaro@gaslini.org (R.C.); federicocresta@gaslini.org (F.C.); ciprandiriccardo@gmail.com (R.C.); 5 Department of Health Sciences, Section of Biostatistics, University of Genoa, 16132 Genoa, Italy; irene.schiavetti@unige.it; 6 Department of Psychiatry, Faculty of Medicine, Geneva University (UNIGE), 1211 Geneva, Switzerland; alessandra.costanza@unige.ch; 7 Department of Psychiatry, Adult Psychiatry Service (SPA), University Hospitals of Geneva (HUG), 1211 Geneva, Switzerland; 8 Department of Psychiatry, Faculty of Biomedical Sciences, University of Italian Switzerland (USI), 6900 Lugano, Switzerland

**Keywords:** temperament, Cystic Fibrosis, CFTR-modulator therapy

## Abstract

The aim of this study was to investigate the association between Cystic Fibrosis (CF) and affective temperaments, considering the relevance of ionic balances in neural excitability, as a possible neurobiological basis for temperamental expression. A cross-sectional study involving 55 adult CF patients was conducted. Sociodemographic, clinical and therapeutic characteristics, temperamental and personality dispositions and depressive and anxiety symptoms were evaluated through standardized semi-structured and structured interviews. The majority of the enrolled CF patients were receiving Cystic Fibrosis Transmembrane Conductance Regulator (CFTR) therapy (72.7%), and most of them had hyperthymic temperament predominance (29.1%). Different TEMPS-A (Temperament Evaluation of Memphis, Pisa, Paris, and San Diego Autoquestionnaire) dimensions were not associated with the type of CF phenotype-related mutation or with the use of CFTR-modulator therapy. However, a tendency towards irritability was noted in patients not undergoing CFTR modulator therapy (6.7 ± 4.72 vs. 4.7 ± 4.33; *p* = 0.13). In light of the limitations imposed by the cross-sectional nature of the study, a hyperthymic temperament was found to be protective against current or lifetime psychopathologic events, whereas the other temperaments were associated with positive psychopathological anamnesis. Based on the measurement of temperament profiles and the study of their associations with clinically relevant variables, we argue that subjecting CF patients to such a temperament assessment could prove beneficial in the transition towards integrated and personalized care.

## 1. Introduction

The concept of temperament dates back further than two thousand years, with its roots tracing back to Hindu sacred texts [1]. In Mesopotamian, Greek and Roman sources, temperament is conceived as a constant tendency of the individual to respond to stimuli in a certain way and is seen as originating from a more or less balanced mixture of bodily humours (in Hippocratic theory: blood, yellow and black bile and phlegm) and correlating with certain physical phenotypes, thus indicating the concept’s deep psychosomatic implications [2,3].

More recently, temperament has generally been defined as constitutionally based individual differences in reactivity and self-regulation influenced over time by genes, maturation and experience [4], and it is seen as the closest neuro-biological correlate mediating the interactions of heredity with environmental and relational experiences in the development of personality from the first years of life [5,6]. Among other definitions of temperament, Akiskal proposed the characterization of “affective temperaments” as hyperthymic (hyp), anxious (anx), dysthymic (dys), cyclothymic (cyc) and irritable (irr), which is largely based on the works of Schneider [7] and Kraepelin [8].

In contrast to categorical bipolar disorders, affective temperaments are continuous, trait-like expressions of affect that are believed to underlie mood psychopathology [9]. A continuum between affective temperaments and mood or anxiety disorders is already under discussion [10]. Evidence from the literature shows that different affective temperament types represent, on the one hand, the subclinical manifestations and, frequently, the precursors of bipolar or depressive disorders, and on the other hand, the predictors of the clinical evolution of major mood disorders, including the direction of polarity and symptom formation of acute affective episodes, thus affecting the long-term course and outcome [11]. In particular, hyperthymic and, to a lesser extent, cyclothymic temperaments seem to be characteristic of bipolar disorder (BD) type I, whereas a dysthymic temperament prevails in depressive disorders [12]. Moreover, dysthymic, cyclothymic, irritable and anxious temperaments are significantly higher in bipolar patients with mixed episodes, while a cyclothymic temperament can also act as a contributing factor in the development of atypical features (appetite and weight gain, hypersomnia, leaden paralysis, interpersonal rejection sensitivity, etc.) in the framework of a major depressive episode [13].

Therefore, affective temperaments seem to have a pathoplastic role in influencing the manifestation and course of illness and the response to therapy for affective disorders [13], as well as the suicide risk in these clinical populations [14,15]. The relevance of a premorbid temperament in delineating the risk of developing non-mood psychopathological events or disorders has also emerged [16].

Far from being a simple passive receptor of stimuli, the nervous system semi-spontaneously/spontaneously expresses hierarchically organized bioelectrical activity, which continuously informs sensory data [17,18,19]. This basic disposition of the system to environmental inputs could represent the biological correlate of the fundamental expression of affectivity. The harmonic orchestration of ionic transits on the neuronal level may be seen as the original building block of this complex and its dynamic arrangement.

Evidence from literature suggests that the dysregulation of ion fluxes leads to unbalanced neural excitability in affective disorders, and several mood stabilizers act on sodium, calcium and potassium conductance [20]. Given the positive correlation of cyclothymic temperament expression with long-term lithium therapy, it stands to reason that a pathology influencing transmembrane dynamics could influence affectivity in general, from temperament expression to major disorders [21,22].

Cystic Fibrosis (CF) is a heritable autosomal recessive pathology caused by the malfunction of a chloride transporter, Cystic Fibrosis Transmembrane Conductance Regulator (CFTR), which causes lung impairment. Improvements in the management strategies and additional therapeutic options have rendered CF a chronic pathology mostly affecting adults, with increasing life expectancy. The recent introduction of CFTR modulator therapy—e.g., Ivacaftor, Elexacaftor, Tezacaftor and Lumacaftor—is contributing to efforts to significantly alter the main epidemiologic variables [23,24].

Mental health issues in CF patients and their caregivers, the most common being depressive and anxious symptoms [25], have been shown to occur more frequently than in the general population by approximately 20%, becoming more likely as patients age [26] and affecting adherence to therapy [27] and, thus, life expectancy [28]. The increased prevalence of mental health issues is usually attributed to the chronic and disabling nature of the disease, but at the moment, this is simply a plausible assumption that should not necessarily be taken for granted.

The aim of this study was to investigate the association between CF and affective temperaments, considering the relevance of ionic balances in neural excitability, as a possible neurobiological basis for temperamental expression.

## 2. Materials and Methods

### 2.1. Participants

The study, as part of a broader research project entitled “Mental Health in Cystic Fibrosis patients: the prognostic role of temperament, personality and attachment styles”, supported by the Italian Cystic Fibrosis Research Foundation (FFC 21/2021) [29], included patients admitted to the CF Center, IRCCS Istituto Giannina Gaslini—Ospedale Pediatrico, Genoa, Italy, recruited between September 2021 and September 2022. The Ethical Committee of IRCCS Istituto Giannina Gaslini—Ospedale Pediatrico approved the study (Registro CER Liguria: 191/2021—DB ID 11351, 29 June 2021), and all the participants gave written informed consent.

The sample size was not calculated a priori, as this study can be considered as an exploratory analysis without a primary hypothesis to be tested. Therefore, all patients admitted to the CF Center within one year of observation who met all the inclusion criteria were enrolled. Patients were enrolled in the study not only based on their fit with the inclusion criteria but also on the basis of their personal willingness to participate. The clinical researchers of the CF Center paid particular attention to this aspect, given the area of investigation, which refers to personological aspects. It was essential to provide the patients with the reasons for the study, making them aware of the intended research purposes.

A CF diagnosis was formulated starting with the newborn screening test for 8 patients and based on suspected symptoms for 47 patients. Sweat testing and genetic testing were used for all the patients enrolled (*n* = 55) to confirm the CF diagnosis. Regarding the age of diagnosis (excluding patients undergoing newborn screening), 38 patients were diagnosed at pediatric age (1–12 y.o.), with 3 patients in adolescence (15–17 y.o.) and only 6 patients in adulthood.

All demographic and clinical information was extracted from a dedicated web-based platform. Descriptive data were presented as the mean with standard deviation (SD), the median with the range or interquartile range (IQR) was used for continuous variables, and a number with the percentage was used for categorical variables, as is appropriate.

The inclusion criteria were as follows: a CF diagnosis, age ≥ 18 years, IQ ≥ 70, a good understanding of the spoken and written Italian language and ability to give informed consent. Patients with clinically relevant neurological conditions were excluded.

Current or lifetime psychiatric diagnoses were made by trained clinical researchers of the CF Center through a non-structured interview according to the Diagnostic and Statistical Manual for Mental Disorders—Fifth Edition (DSM-5) criteria [30]. Standardized semi-structured and structured interviews were used to assess the sociodemographic, clinical and therapeutic characteristics, temperament and personality dispositions and depressive and anxiety symptoms. Temperament predominance was determined on the basis of a higher Z-score dimensional deviation of the subject according to the Temperament Evaluation of Memphis, Pisa, Paris and San Diego Autoquestionnaire (TEMPS-A) relative to the mean sample dimensional values.

### 2.2. Assessment Scales

The study included the administration of the following standardized questionnaires:

- The Temperament Evaluation of Memphis, Pisa, Paris and San Diego Autoquestionnaire (TEMPS-A), Italian-validated version [31], including 110 items, to assess temperament characteristics (dysthymic, cyclothymic, hyperthymic, irritable and anxious). The chosen scoring system consisted of assigning 1 point for each positive answer and 0 points for negative answers within the sub-scales (dysthymic items 1–22, cyclothymic items 23–42, hyperthymic items 43–63, irritable items 64–84 and anxious items 85–110) and then calculating the dimensional sums. Item 84 asks specifically about temperament before menstrual cycles and was designated for women only. The Italian version of the TEMPS-A was validated based on a sample of 948 nonclinical subjects (27.39 years ± 8.22 S.D.), including 476 men (50.2%: 28.56 years ± 8.63 S.D.) and 472 women (49.8%: 26.21 years ± 7.61 S.D.). The findings were consistent with those of TEMPS-A studies from different countries. The reliability of the TEMPS-A was assessed using the Cronbach alpha coefficients for the components, and they were quite high; the alpha computed for the first subscale, with the largest number of items, was 0.89, while that for the irritable subscale was 0.77 and that for the hyperthymic subscale was 0.74.
- The Minnesota Multiphasic Personality Inventory-II Restructured Form (MMPI-2-RF), Italian-validated version [32], to evaluate personality characteristics. The MMPI-2-RF is a 338-item (true/false), multiscale, self-report inventory that measures a wide range of psychopathology symptoms and maladaptive personality traits. The 338 MMPI-2-RF items are aggregated onto fifty-one individual scales. Nine of these, the Validity Scales, measure various forms of response styles that, when excessive, could invalidate a test protocol. The remaining forty-two scales measure substantive clinical contents. The three Higher-Order Scales, including Emotional/Internalizing Dysfunction, Thought Dysfunction and Behavioral/Externalizing Dysfunction, index broadband psychopathology constructs of, respectively, internalizing, thought disorder and externalizing. The nine Restructured Clinical Scales reflect transdiagnostic dimensional psychological constructs rather than psychiatric syndromes. The twenty-three Specific Problems Scales, the most narrowband symptom and trait measures in the instrument, are organized into four thematic domains: Somatic/Cognitive, Internalizing, Externalizing and Interpersonal, which also reflect the general interpretive organization of the instrument. The two Interest Scales (Aesthetic–Literary and Mechanical–Physical) primarily measure personality and attitudinal constructs rather than clinical symptoms or traits. Finally, the five Personality Psychopathology Scales are revised versions of their MMPI-2 counterparts, representing dimensional personality traits with an abnormal range and presented as a dimensional alternative to the categorical personality disorder framework that dominates the DSM.
- The Patient Health Questionnaire (PHQ-9), Italian-validated version [33], to assess depressive symptoms. The PHQ-9 is a 9-item self-report measure for assessing and screening the severity of depressive symptoms. For each item, patients are asked to assess how much they have been bothered by symptoms over the last 2 weeks. There are 4 answer options: not at all (0), several days (1), more than half of the days (2) and nearly every day (3). The sum score (range 0–27) indicates the degree of depression, with scores of ≥5, ≥10 and ≥15 representing mild, moderate and severe levels of depression.
- The General Anxiety Disorder (GAD-7) questionnaire to assess anxiety symptoms [34]. The GAD-7 is a 7-item self-report measure for assessing and screening generalized anxiety disorder and its severity during the past 2 weeks. The items are rated on a 4-point Likert scale ranging from 0 (‘Not at all’) to 3 (‘Nearly every day’), with a total score ranging from 0 to 21. Higher scores indicate higher levels of generalized anxiety. Scores ranging from 10 to 14 indicate generalized anxiety of moderate severity, and scores ranging from 15 to 21 indicate severe generalized anxiety. For our study, we used the official Italian version, which is freely downloadable from the PHQ website (http://www.phqscreeners.com, accessed on 4 April 2023).

### 2.3. Statistical Analysis

Due to the nature of the data, mostly non-parametric testing was used (e.g., Spearman’s rho correlation, Mann–Whitney U test, Kruskal–Wallis test). The TEMPS-A scores were presented in terms of Z-scores, considering the mean and standard deviation of both the sample and data acquired from the literature [34,35]. For each temperament dimension, a comparison between the Z-scores of different sets (the sample population, the general population sample and clinical BD type I and II and major depressive disorder (MDD) samples) was performed with a non-parametric variance analysis (Friedman’s two-way analysis of variance by rank for related samples) and subsequent Bonferroni’s correction for a pairwise comparison. The prominence of the different temperament dimensions was defined for each subject based on the highest variance from the sample mean values relative to the single temperament measurements.

Statistical significance was set as α ≤ 0.05. The statistical analysis was conducted using the SPSS Statistical package, version 24.0. (IBM Corp, Armonk, NY, USA).

## 3. Results

The baseline characteristics of the sample are presented in Table 1. In the evaluation of affective temperaments among the adult CF population (*n* = 55; age = 34.3 ± 10.9 years; F = 70.9%), the majority of whom were receiving CFTR modulation therapy (*n* = 40; 72.7%), we recorded *n* = 34 (61.8 %) cases of a current or lifetime psychiatric diagnosis, observed mainly in the female population (*n* = 27/39; 69.2%), with the most frequent diagnoses being anxiety disorder (*n* = 27) followed by cyclothymic disorder (*n* = 6). Major psychiatric diagnoses less often referred to BD (*n* = 1) or MDD (*n* = 3). Approximately 85% of the sample (*n* = 20) had a positive family history for psychiatric disorders. Diabetes was the main medical comorbidity, as expected, in the adult CF population.

**Figure 1:**
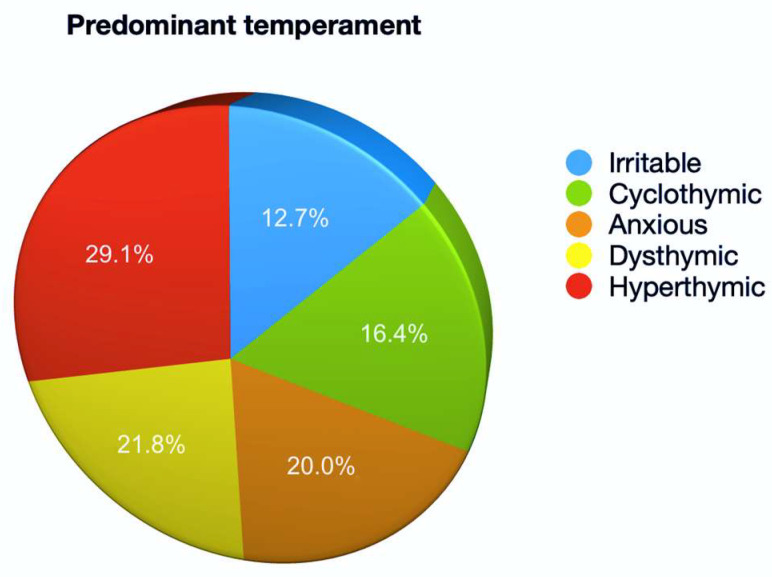
Sample Z-score temperament distribution (N = 55).

The study sample showed a predominance of hyperthymic temperaments (29.1%) (Figure 1). No TEMPS-A dimension was correlated with age, whereas a statistically significant difference in anxious temperament scores was observed between the sexes (males: 9.7 ± 5.94 vs. females: 6.1 ± 5.74; *p* = 0.029), and higher values for cyclothymic and dysthymic traits were observed in females compared to males, without fully reaching statistical significance.

Different TEMPS-A dimensions were not associated with the category of CF phenotype-associated mutation or with the use of CFTR modulator therapy. However, although not statistically significant, a tendency towards irritability was registered in patients not undergoing CFTR modulator therapy (6.7 ± 4.72 vs. 4.7 ± 4.33; *p* = 0.13). An irritable temperament was also significantly associated with a cyclothymic temperament (rho = 0.719, *p* < 0.001).

The correlation analysis between different TEMPS-A dimensions indicated a moderate/strong relationship (and then a co-expression) between the following temperaments: cyclothymic–dysthymic (rho = 0.64; *p* < 0.001); dysthymic–irritable (rho = 0.48; *p* < 0.001); dysthymic–anxious (rho = 0.53; *p* < 0.001); cyclothymic–irritable (rho = 0.72; *p* < 0.001); cyclothymic–anxious (rho = 0.65; *p* < 0.001) and irritable–anxious (rho = 0.65; *p* < 0.001). The hyperthymic dimension was not correlated with the any of the others.

**Figure 2:**
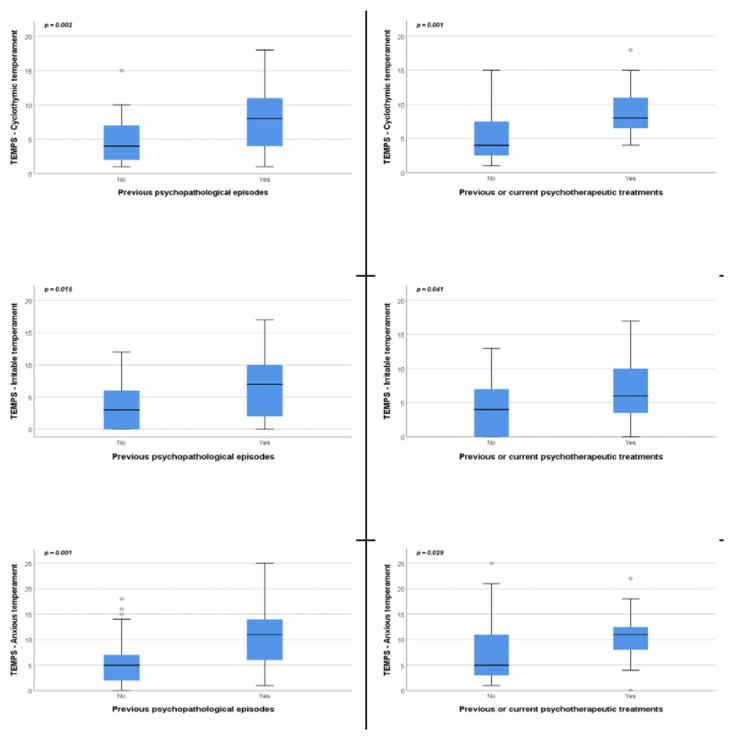
Significant TEMPS-A—psychiatric anamnesis correlations. TEMPS-A: Temperament Evaluation of Memphis, Pisa, Paris and San Diego Autoquestionnaire. Hollow dots represent high or low potential outliers. In the boxplot, top line is the Q3, middle line is Q2, bottom line is Q1, and upper (and lower) whisker extend to maximum (and minimum) data point within 1.5 box heights from top (or bottom) of the box.

Differences in the cyclothymic, irritable and anxious domains were found between patients with and without previous psychopathological episodes and between patients with or without prior psychologic or psychotherapeutic treatment (Figure 2). No difference was found between those who used and did not use psychopharmacologic therapy.

Patients with a family history positive for psychiatric illnesses showed higher cyclothymic and anxious temperament scores compared to those with a negative history (8.4 ± 4.30 vs. 5.7 ± 4.12, respectively, *p* = 0.021, for cyclothymic temperament; 11.7 ± 5.90 vs. 7.0 ± 5.54, respectively, *p* = 0.005, for anxious temperament). 

**Figure 3:**
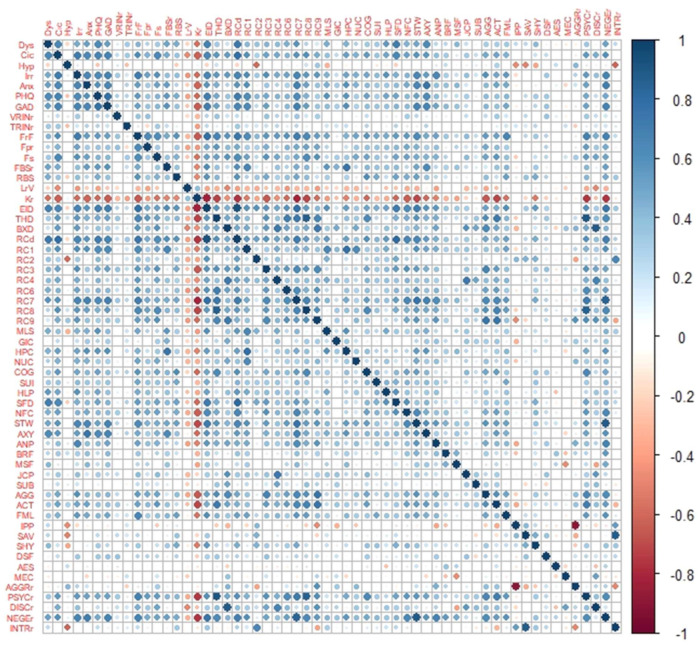
TEMPS–A, PHQ–9, GAD-7 and MMPI–2–RF score correlation matrix. TEMPS–A: Temperament Evaluation of Memphis, Pisa, Paris and San Diego Autoquestionnaire; PHQ–9: Patient Health Questionnaire; GAD–7: General Anxiety Disorder; MMPI–2–RF: Minnesota Multiphasic Personality Inventory–II Restructured Form; DYS: dysthymic; CYC: cyclothymic; HYP: hyperthymic; IRR: irritable; ANX: anxious; VRINr: variable response inconsistency; TRINr: true response inconsistency; FrF: infrequent responses; Fpr: infrequent psychopathology responses; Fs: infrequent somatic responses; FBSr: symptom validity; RBS: response bias scale; LrV: uncommon virtues; Kr: adjustment validity; EID: Emotional/Internalizing Dysfunction; THD: Thought Dysfunction; BXD: Behavioral Externalizing Dysfunction; RCd: demoralization; RC1: somatic complaints; RC2: low positive emotions; RC3: cynicism; RC4: antisocial behavior; RC6: ideas of persecution; RC7: dysfunctional negative emotions; RC8: aberrant experiences; RC9: hypomaniac activation; MLS: malaise; GIC: gastrointestinal complaints; HPC: head pain complaints; NUC, neurological complaints; COG: cognitive complaints; SUI: suicidal death ideation; HLP: helplessness, hopelessness; SFD: self-doubts; NFC: inefficacy; STW: stress, worry; ANX: anxiety; ANP: anger proneness; BRF: behavior-restricting fears; MSF: multiple specific fears; FML: family problems; IPP: interpersonal passivity; AV: social avoidance; SHY: shyness; DSF: disaffiliativeness; AES: aesthetic–literary interests; MEC: mechanical-physical interests; AGGR–r: aggressiveness; PSYC–r: psychoticism; DISC–r: discontraint; NEGE–r: negative emotionality, neuroticism; INTR–r: introversion, low positive emotionality.

A correlation matrix evaluating the relationships between each TEMPS-A dimension and further variables of interest, including the PHQ-9 and GAD-7 scores and MMPI-2-RF dimension T values, is shown in Figure 3.

**Figure 4:**
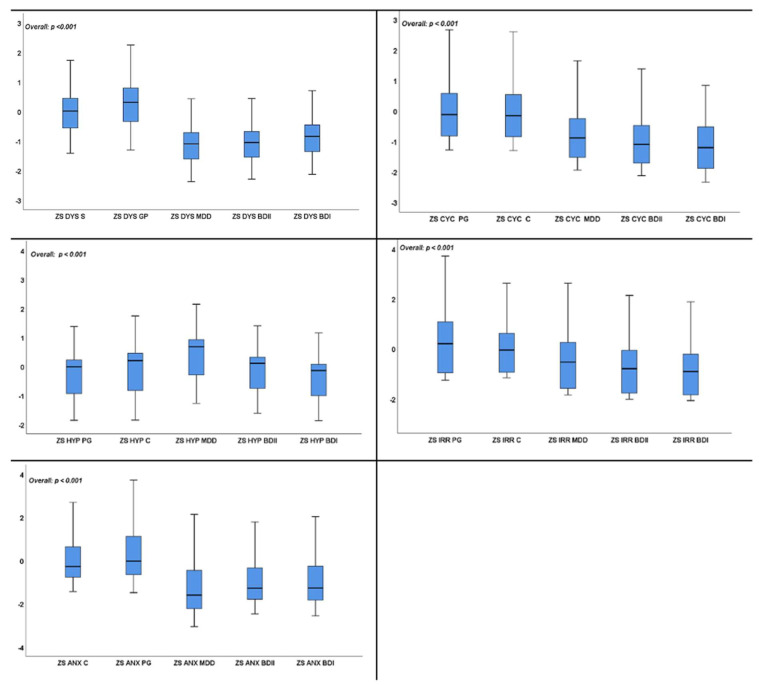
Multiple–Z–score comparative analysis. ZS: zeta score; HYP: hyperthymic; DYS: dysthymic; ANX: anxious; CYC: cyclothymic; IRR: irritable; GP: general population; S: sample; MDD: major depressive disorder; BDI: bipolar disorder type I; BDII: bipolar disorder type II.

Comparisons of the TEMPS-A temperaments between the different analytical sets are reported in Figure 4. For all the traits, the median Z-score result differed between sets.

## 4. Discussion

We examined a sample of adult CF patients considering their demographic and psychopathological variables, thus confirming literature data documenting the frequent experience of psychiatric symptoms among CF patients, predominantly women, who are most often affected anxiety disorders, with only a few possessing a major psychiatric diagnosis. In patients with a positive family history for general psychopathology, cyclothymic and anxious temperaments were more frequently expressed. The majority of patients were receiving CFTR therapy and, oddly, most of them expressed a predominant hyperthymic temperament dimension. No TEMPS-A dimension was correlated with age, whereas a statistically significant difference in anxious temperament scores was observed between the sexes (higher in males compared to females), and higher values for cyclothymic and dysthymic traits were observed in females compared to males, albeit without fully reaching statistical significance.

**Figure 5:**
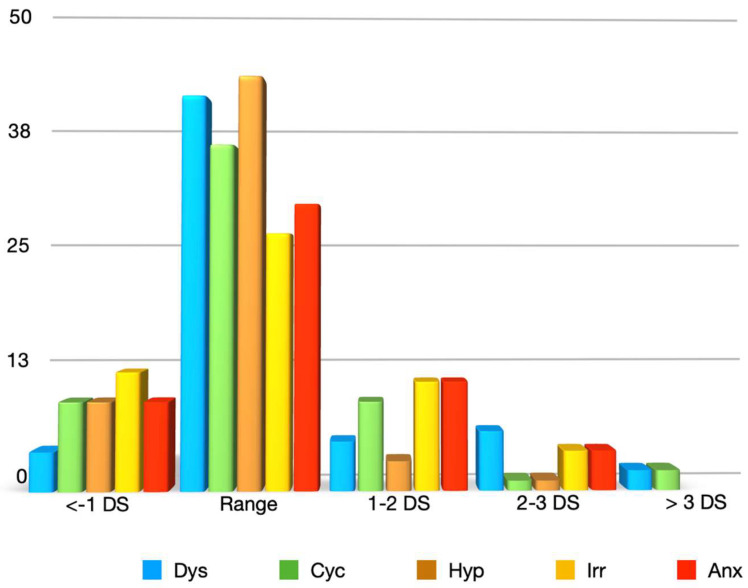
Variance in the temperament scores compared to the mean scores of a large general population sample [35]. DYS: dysthymic; CYC: cyclothymic; HYP: hyperthymic; IRR: irritable; ANX: anxious.

The variance in the temperament scores, when compared to the mean scores of a large general population sample [35], showed roughly symmetrical distribution diagrams (Figure 5), except for the hyperthymic temperament. The hyperthymic temperament scores were inferior in our sample, with a high negative variance, probably due to sampling discrepancies in the chosen general population which favored young males, who are known to frequently express hyperthymic trait dominance [36]. In the subsequent Z-score comparison of the clinical population samples (BD type I and II, MDD) [37], being more congruent to our study population championing-wise, the hyperthymic temperament distribution in our sample was found to be more similar to that of the BD type II population.

The correlation matrix (Figure 3) highlights other relevant associations. Higher psychopathological scores (PHQ-9, GAD-7, MMPI-2-RF), capturing a picture of present-moment suffering, are significantly correlated with the fourfold cluster of dysthymic, cyclothymic, irritable and anxious temperaments. Each temperament in this cluster varies in terms of the strength of its link to each associated variable; for instance, the dysthymic temperament was linked to a higher PHQ-9 score, i.e., to depressive symptoms. The temperament expression patterns show cyclothymic, irritable and anxious to be correlated with the number and prevalence of prior psychopathological episodes and current or past psychotherapeutic treatment. Moreover, anxious and cyclothymic temperaments were significantly correlated with psychiatric familiarity, and the most frequent diagnoses were anxiety and cyclothymic disorders. Lastly, all the temperament dimensions except for the hyperthymic were significantly associated with the MMPI-2-RF psychosomatic symptom score, which brings us back to the corporeity of temperament expression, made especially relevant here by the debilitating nature of the CF disease.

The fourfold temperament cluster predominance was recently defined as typical of the proposed “cyclothymic-sensitive” subgroup of bipolar populations, who, in this case, were more frequently females and reported higher number of depressive, hypomanic and suicide attempts when compared to the dominantly hyperthymic patients. On the contrary, these latter patients showed a higher number of manic episodes and hospitalizations than the cyclothymic-sensitive patients [38], underlining the link between said cluster and both present and past, familiar psychiatric symptoms.

Only the hyperthymic temperament fell short of correlating with the prevalence of prior psychopathologic events or the MMPI-2-RF, PHQ-9 and GAD-7 scores, confirming it as a possible protective factor: what we found was not simply an absence of correlation with the psychopathologic burden but, in some cases, an inverse correlation.

Evidence from the literature describe a hyperthymic temperament as being associated with significant genetic loading (i.e., chromosomes 12 and 22; genes MDM1 and FBLN1) [39,40], leading to the hypothesis that it could represent an evolutionary adaptive advantage [41,42]. A hyperthymic temperament plays a protective role against the development of mental illnesses [43] and suicide risk in clinical populations [44,45], among whom it tends to predict better outcomes of manic episodes [46] and overall improved outcomes of BD and MDD [47,48]. At the same time, hyperthymic temperament dominance represents a risk factor for the number/severity of manic episodes in BD [38], as well as mixed events [49]. In accordance with this vulnerability profile, Akiskal proposed the use of a fourth bipolar subtype, BD-IV, to describe cases of hyperthymic temperament/trait hypomania underlying a prevalent depressive recurrence [50]. 

The possible protective role of hyperthymic temperaments was confirmed in our study, both indirectly through the absence of a correlation with psychopathologic events and directly through a comparison of the MMPI-2-RF scores, i.e., they are positively correlated with the MMPI-2-RF construct of “K-r adaptation validity” (*p* = 0.103) and negatively with items such as low positive emotions and social avoidance [51].

Within specific populations, the overexpression of hyperthymic temperament traits as either constitutively or pharmacologically favored (e.g., lithium therapy; 5HT-agonists, etc.) [52,53], could lead to adverse consequences, e.g., in subjects with undiagnosed mood disorders at risk of presenting mixed mood episodes [50,54,55], which, in turn, is correlated with a higher suicide risk [56,57,58,59,60,61].

Although, within our sample, CFTR modulator therapy had no impact on temperament expression (apart from a tendency towards irritability in non-drug-treated subjects), in terms of absolute numbers, a hyperthymic temperament resulted disproportionately more numerous cases in our CFTR therapy subpopulation, who had similar or lower scores than the non-drug-treated patients for the other four dimensions.

This could point to a negative modulatory effect of CFTR-targeted therapy on cluster expression, as seen with the effect of lithium therapy in a comparable bipolar sample [22], i.e., long-term lithium was correlated with a prevalence of hyperthymic temperament expression. On the one hand, if the harmony of ionic currents is the neurobiological basis for affective and temperament expression, and if CF patients are at risk of developing affective disorders because of disturbances in these harmonies, then an ion-current-targeted drug could help to restore the balance and possibly play a protective role against psychopathological events. On the other hand, some “at-risk” conditions could be better identified before the start of therapy with CFTR modulators.

This mechanism could help to explain some of the most severe adverse events, including suicidal ideation and attempts, recorded in the first clinical trials of CFTR modulation therapy [62,63], considering, in addition, the association between a hyperthymic temperament and MMPI-2-RF variables of activation/aggression. It should be noted that the worsening of anxious–depressive symptoms and the occurrence of passive and active suicidal ideation, according to the literature, were observed to occur most frequently in subjects undergoing Lumacaftor/Ivacaftor treatment who presented with the following at-risk characteristics: female sex, adolescent age, positive psychiatric anamnesis and often-untreated previous anxious–depressive feelings [62,63].

Further studies should investigate the role of CFTR modulator therapy in mental health outcomes to better understand how the modulation of ion channels, touching on neuronal dynamics and excitability, coincide with the altered expression of temperament traits.

This study should be interpreted in the light of several limitations. Firstly, the small sample size did not allow us to study the correlation between temperamental expression and the specific mutation type. Secondly, although without direct implications, the methodological adequacy was limited by the fact that the sample used was a subpopulation of a wider study with a different scope [29]. Moreover, some of the possible implications (i.e., the impacts CFTR-targeted therapy on cluster expression and subsequent processes) are based on data differences that are not statistically significant. Although, in our opinion, such evidence could be confirmed with adequate statistical power in further studies, to date, we have only a hypothesis that is not fully supported. At the same time, the cross-sectional nature of our study limits the value of its implications related to the psychiatric history of the patient. Lastly, the general population sample, which we chose due to its numerosity and accuracy, resulted in some confounding factors. As a direct consequence, the hyperthymic temperament scores were inferior in our sample, with a high negative variance, probably due to sampling discrepancies in the chosen general population which favored young males, who are known to frequently express hyperthymic trait dominance [36]. It is likely that with a general population sample more congruent to our study population championing-wise, the hyperthymic temperament distribution in our sample would be found to be more similar to that of a BD type population.

## 5. Conclusions

To the best of our knowledge, this was the first study that assessed temperament traits in CF patients, although data from the literature strongly argue for a significant association between CF and mental health issues. To date, this evidence led to the establishment of the International Committee on Mental Health in CF patients, which recommend PHQ-9 and GAD-7 as the best tools for screening and monitoring the treatment response. In our opinion, because of the complexity of CF and its potential mental health implications, more attention should be paid to potential risk factors that could be used as potential prime targets for interventions in the context of later psychiatric disorders. We focused on temperament as a potential risk factor for later mood and anxiety disorders, and we investigated the associations between temperament traits and CF, considering the relevance of ionic balances in neural excitability, as a possible neurobiological basis for temperamental expression.

Through the measurement of temperament profiles and the study of their correlations with clinically relevant variables, we found that subjecting CF patients to such a temperament assessment can prove beneficial in the transition towards integrated and personalized care. Transitioning to primary prevention strategies could enable the early recognition of subjects at risk of affective symptoms or even major psychiatric diagnoses, which could, in turn, negatively impact on the patient’s quality of life, life expectancy and compliance with therapy.

Moreover, further studies are needed to better explore the relationship between CFTR modulation therapy and temperament expression. On the one hand, most of the sample population expressed a dysthymic, cyclothymic, irritable and anxious cluster predominance, which seemed to be related to psychopathologic suffering, which was over-represented in our sample compared to the general population. On the other hand, almost all the drug-treated subjects and subjects with drug-sensible mutations had higher hyperthymic TEMPS-A scores and lower scores in the cluster dimensions; thus, the expression of a hyperthymic temperament seemed to be related to generally lesser suffering, expressed in terms of MMPI-2-RF, GAD-7 or PHQ-9 scores. Despite all the indications of a possible protective role of hyperthymic temperaments against psychopathology, and the as-yet unexamined hypothesis of hyperthymic trait upregulation in patients undergoing ion transporter therapy, caution should be exercised given the different subpopulations, together with their risk profiles (e.g., cyclothymic-sensitive and BD-IV), and the built-in propensity for activation among subjects expressing predominant hyperthymic traits, being well-documented in cases of increased activation, aggression and even suicide in clinical populations undergoing CFTR-targeted therapy [63].

## Figures and Tables

**Table 1:** Demographic and clinical–anamnestic data (N = 55).

		Total	Female	Male
Age		34.3 ± 10.94 (19.0−60.0)	34.9 ± 12.07 (19.0−60.0)	32.9 ± 7.65 (22.0−48.0)
Gender	Female	39 (70.9%)	39 (100.0%)	0 (0.0%)
	Male	16 (29.1%)	0 (0.0%)	16 (100.0%)
CFTR modulation therapy		40 (72.7%)	31 (79.5%)	9 (56.3%)
Prior psychopathologic episodes		30 (54.5%)	26 (66.7%)	4 (25.0%)
Number of prior psychopathologic episodes		2.2 ± 0.92 (0.0−4.0)	2.2 ± 0.98 (0.0−4.0)	2.3 ± 0.50 (2.0−3.0)
Presence of at least one psychiatric diagnosis		34 (61.8%)	27 (69.2%)	7 (43.8%)
Anxiety disorder		27 (49.1%)	21 (53.8%)	6 (37.5%)
Depressive disorder		3 (5.5%)	2 (5.1%)	1 (6.3%)
Cyclothymic mood disorder		6 (10.9%)	6 (15.4%)	0 (0.0%)
Dissociative disorder		1 (1.8%)	1 (2.6%)	0 (0.0%)
Dysthymic disorder		9 (16.4%)	5 (12.8%)	4 (25.0%)
Obsessive compulsive disorder		1 (1.8%)	0 (0.0%)	1 (6.3%)
Bipolar disorder		1 (1.8%)	1 (2.6%)	0 (0.0%)
Eating disorder		1 (1.8%)	1 (2.6%)	0 (0.0%)

## Data Availability

The data that support the findings of this study and its materials are available from the corresponding author upon request.
